# Cognitive mechanisms and neurological foundations of companion animals’ role in enhancing human psychological well-being

**DOI:** 10.3389/fpsyg.2024.1354220

**Published:** 2024-04-24

**Authors:** Heng Liu, Jingyuan Lin, Wuji Lin

**Affiliations:** Institute of Brain and Psychological Sciences, Sichuan Normal University, Chengdu, China

**Keywords:** human-animal interaction, animal-assisted therapy, companion animals, cognitive neural mechanisms, emotion regulation

## Abstract

The impact of companion animals on human psychological health has garnered widespread attention. Research demonstrates that companion animals contribute positively in various ways, including reducing depression, anxiety, stress, and fostering positive emotions in humans. Recent studies have revealed significant changes in the activity levels of human emotion-related cortical areas (such as the frontal cortex and amygdala) and neurotransmitter (e.g., oxytocin, cortisol) secretion due to interaction with companion animals. However, research in this domain is still in a nascent stage, with many unknowns in the cognitive neural mechanisms involved. This paper proposes that to understand the cognitive mechanisms through which companion animals affect human psychological health, we need to examine changes in emotional cognitive processing. It aims to uncover the neurological underpinnings of how companion animals enhance human psychological well-being from the perspective of brain connectivity. This approach is expected to provide theoretical support and direction for future research and practical applications in this field.

## 1 Introduction

“Why are humans so fascinated by cats and dogs?” was highlighted as a prime scientific inquiry by Science in 2021 (Sanders, 2021). The bond between humans and companion animals, extending over millennia, includes documented instances of companion animals enhancing human health from the 19th century onwards. Nightingale noted the beneficial impact of pet birds on patient health (Nimer and Lundahl, 2007). Recent studies indicate that companion animals contribute to the improvement of human physiological and psychological well-being (Wells, 2019), demonstrating parallel patterns of attachment in both behavior and physiology between companion animals and humans (Zilcha-Mano et al., 2011; Solomon et al., 2019). Forming and sustaining this attachment might entail intricate biological processes like neural regulation and hormonal secretion, crucial for comprehending the influence of companion animals on human psychological health (Julius et al., 2012). While the beneficial impact of companion animals on human psychological health is partly validated and broadly acknowledged, the cognitive mechanisms and fundamental neural processes involved are not fully understood and necessitate additional research (Borgi and Cirulli, 2016; Beetz, 2017). Consequently, this paper aims to consolidate and review current literature in this domain, addressing the effects of companion animals on human psychological health and the recent cognitive and neural research, thereby enriching the understanding of the mechanisms underlying human-animal interactions.

## 2 Bibliometric analysis of the relationship between companion animals and human psychological health

The question, “What is the relationship between companion animals and human psychological health?” has long been a focus of extensive research interest, with scholarly output increasing year by year (Yatcilla, 2021). This paper begins by employing bibliometric analysis to quantitatively present the current research achievements in this field, thereby revealing the developmental trends of this area of study. We must clarify that the search method and results are exclusively for bibliometric analysis and do not represent the paper’s entire research foundation. Our review extends beyond these search results, although they are included.

**Figure 1:**
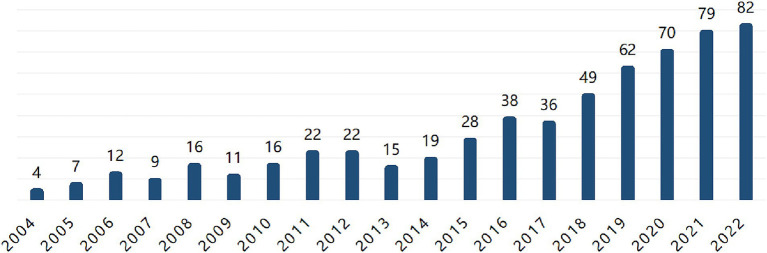
Distribution of the number of publications from 2004 to 2022.

The analysis utilized data from the Web of Science database covering the years 1982 to 2022, employing search terms: [TS = (“pet” or “companion animal” or “pets” or “companion animals” or “human-animal interaction” or “human-animal interactions” or “human-animal bond”)] and [TS = (“psychology” or “psychological” or “mental health” or “animal-assisted therapy” or “psychic” or “psychosis” or “psychiatry”)]. From the 2,804 articles initially retrieved, 597 papers (from 2004 to 2022) relevant to this study’s theme were filtered and analyzed using CiteSpace 6.2.R2 software. The analysis revealed a rising trend in the annual number of publications from 2004 to 2022 (see Figure 1), corroborating previous findings (Yatcilla, 2021).

An analysis of the 597 papers identified 418 unique keywords. Among them, 20 word groups appeared more than 45 times (see Table 1). Prominently, keywords related to “Animal assisted therapy” and “Pet therapy” were frequently observed, underscoring their significance in the field. Animal-assisted therapy, a crucial application in human-animal interaction, has a long-standing history and has evolved into a mature psychological treatment modality, showing significant effectiveness in groups like patients with emotional disorders, special needs children, and prisoners (Nimer and Lundahl, 2007; Liu and Gao, 2021; Wang and Liu, 2021). The term “Dog (s)” also featured prominently, partly because dogs are the most commonly used animals in animal-assisted therapy research (Chandler, 2001; Dimitrijević, 2009) and partly due to recent findings that different types of animals have varying impacts on humans (Hajek and König, 2020; Oliva and Johnston, 2021; Matsumura et al., 2022), indicating a new trend in exploring the differential effects of various animal types. High-frequency keywords also included terms like “Depression” and “Stress,” highlighting the field’s focus on interventions for these negative emotional states. “Attachment” is considered a potential mechanism through which companion animals improve human health (Wells, 2019) and an important modulator of psychological well-being (Peretti, 1990; Miltiades and Shearer, 2011), thus being a frequent subject in studies. Additionally, the impact of companion animals on children’s psychological development, including empathy, self-esteem, and cognitive development (Wilks, 1999; Endenburg and van Lith, 2011; Bachi and Parish-Plass, 2017), is a topic of interest among developmental psychologists. Moreover, the improvement of quality of life (Ortmeyer et al., 2023) and the provision of social support by companion animals are also important areas of focus in this field.

**Table 1:** Keywords frequency and centrality in publications from 2004 to 2022.

Keywords	Frequency	Centrality
Animal assisted therapy	193	0.21
Pet ownership	119	0.09
Dog(s)	115	0.14
Companion animals	104	0.12
Human-animal interaction(s)	99	0.03
Depression	86	0.04
Health	84	0.1
Attachment	75	0.09
Pet(s)	65	0.1
Pet therapy	64	0.05
Mental health	60	0.04
Children	58	0.08
Human health	58	0.05
Behavior	51	0.07
Stress	51	0.03
Physical activity	49	0.02
Quality of life	47	0.04
Social support	47	0.04
People	46	0.08
Ownership	46	0.02

## 3 Studies on companion animals improving human psychological health

Companion animals play a crucial role in both reducing negative emotions and boosting positive emotions in humans. In terms of diminishing negative emotions, companion animals have been shown to significantly alleviate depression (Lem et al., 2013; Ko et al., 2016; Brooks et al., 2018), anxiety (Shiloh et al., 2003; Mossello et al., 2011; Kogan et al., 2021), stress (DeSchriver and Riddick, 1990; Wells, 2005; Trammell, 2017), loneliness (Knight and Edwards, 2008; McConnell et al., 2011; Pikhartova et al., 2014), and other negative emotional states. In enhancing positive emotions, companion animals contribute to directly or indirectly boosting subjective well-being, life satisfaction, and other positive states (Zhou et al., 2020; Curl et al., 2021; Schwarzmüller-Erber et al., 2021; Junça-Silva, 2022). The following summary will concentrate on the principal ways companion animals improve human emotions.

### 3.1 Depression

Numerous studies to date indicate that companion animals significantly alleviate depression, particularly in elderly companion animal owners (Souter and Miller, 2007; Friedmann and Son, 2009; Brooks et al., 2018). For instance, research has uncovered a significant negative correlation between the degree of engagement elderly individuals have with their companion animals and their levels of depression. This effect is particularly pronounced in those living with a spouse and having access to social resources, where high interaction with companion animals has a more notable positive impact on preventing depression (Cheung and Kam, 2018). Another study examined the relationship between companion animal attachment support, human social support, and depression, finding that companion animal attachment support (in contrast to human social support) can effectively reduce or prevent depression in companion animal owners (Krause-Parello, 2012).

Human aging is inevitable, with accompanying loneliness and physical impairments potentially causing significant psychological harm to the elderly. Caring for a companion animal can provide the elderly with a renewed sense of purpose and reason for living, thereby reducing their depression levels and promoting overall physical and mental health (Krause-Parello et al., 2019).

### 3.2 Anxiety

Companion animals also play an effective role in alleviating anxiety. Kogan et al. (2021) conducted an online survey with 5,061 participants, finding that companion animals significantly reduced owner anxiety during the COVID-19 lockdown, particularly among women under 40. Shearer et al. (2016) implemented a four-week intervention involving mindfulness practices and interaction with dogs, noting significant reductions in anxiety levels through both approaches. Hoagwood et al. (2022) incorporated Cognitive Behavioral Therapy (CBT) principles and found that equine-assisted adaptive riding programs had a significant impact on reducing anxiety in adolescents.

Attachment Theory posits that the emotional relationship between humans and companion animals is mutually supportive and beneficial. Companion animals provide unconditional love and support, thereby reducing the incidence of psychological and physiological health problems (Teo and Thomas, 2019). Studies also indicate that companion animals can induce and modulate physiological states of anxiety and arousal, ultimately decreasing anxiety levels in individuals (Berget and Braastad, 2011).

### 3.3 Stress

Severe or prolonged stress can lead to various psychological and physiological problems (Iwata et al., 2013). Companion animals have a beneficial effect on moderating human stress levels (Janssens et al., 2021; Junça-Silva et al., 2022; Rathish et al., 2022), especially for individuals in high-stress work environments (Cevizci et al., 2012). Companion animals often play an important role as attachment figures in human lives (Sable, 2013). Interactions with attachment figures, such as companion animals, in a healthy attachment relationship, can mitigate stress, explaining how interaction with companion animals can reduce stress levels.

Additional research indicates that indirect exposure to companion animals, through viewing images or videos, also mitigates stress (Wells, 2005; Anderson et al., 2017; Ein et al., 2023). Specifically, Ein et al. (2023) observed a reduction in stress levels among participants who watched companion animals videos before undertaking stressful tasks. Anderson et al. (2017) demonstrated that viewing animal-inclusive VR scenes post-stress tasks induced relaxation. Na and Dong (2023) reported that interactions with virtual cats in a mixed-reality (MR) setting effectively diminished psychological stress. This suggests that indirect companion animals interactions may also contribute to stress alleviation.

### 3.4 Positive emotions

In addition to diminishing negative emotions, companion animals significantly boost positive emotions in humans. Studies indicate higher levels of happiness in companion animal owners compared to non-companion animal owners (Bao and Schreer, 2016). Companion animals provide numerous benefits, such as companionship, enhanced social skills, and reduced feelings of loneliness, crucial for increasing life satisfaction (Himsworth and Rock, 2013). Companion animals offer emotional support in stress management, thereby improving psychological adaptability and life satisfaction (Barklam and Felisberti, 2023). companion animal-friendly work environments also positively affect employee well-being (Junça-Silva, 2022). The positive impacts of companion animals extend beyond common companion animals like cats and dogs; other animal types, such as aquarium fish, reptiles, and birds, also contribute significantly to human physical and mental health by enhancing happiness, emotional support, and the pleasure of nurturing life (Riddick, 1985; Kidd and Kidd, 1998; Clements et al., 2019; Azevedo et al., 2022).

Core Conditions Theory posits that humans perceive companion animals as empathetic, nonjudgmental companions who offer unconditional positive attention (Teo and Thomas, 2019). This supportive interaction with companion animals plays a role in regulating emotions, which in turn promotes human psychological health.

### 3.5 Cognition and personality

Beyond emotional aspects, companion animal ownership and human-companion animal interactions also positively influence personality development and cognitive functions (Virues-Ortega et al., 2012). Companion animals encourage the development of children’s confidence and self-esteem (Purewal et al., 2017), while interactions with companion animals enhance empathy, sense of responsibility, social status among peers, and cognitive abilities such as observation, analogy, and reasoning (Melson, 2003; Dimitrijević, 2009; Williams et al., 2010; Endenburg and van Lith, 2011; Pennacchio et al., 2018). Moreover, in medical and rehabilitation settings, companion animals have shown positive impacts on cognitive functions in Alzheimer’s patients, schizophrenia patients, and the elderly (Moretti et al., 2011; Menna et al., 2016; Chang et al., 2021). Interestingly, similar positive effects are observed with robotic companion animals as well (Leng et al., 2019; Pollak et al., 2022).

## 4 Contradictory evidence

While many studies highlight the beneficial impact of companion animals on human mental health, there is also evidence that contradicts these findings. Such studies question the widely accepted notion of a positive link between companion animals and human mental health, suggesting potential adverse effects. The divergent outcomes of these investigations necessitate a prudent approach to comprehending the complex dynamics between companion animals and mental health.

### 4.1 Lack of significant association between companion animals and psychological health

Research suggests the correlation between companion animals ownership and human mental health may not be as robust as once believed. For example, the existence of companion animals shows no substantial link with levels of human depression (Branson et al., 2016; Batty et al., 2017; Hajek and König, 2020), anxiety (Bradley and Bennett, 2015), and stress (Bradley and Bennett, 2015; Cui et al., 2021). Additionally, the beneficial impacts of companion animals on the mental health of children (Miles et al., 2017), the elderly (Wells, 2009), and cancer patients (Ingram and Cohen-Filipic, 2019) remain unverified. Contradictory results across various studies may stem from the widespread use of cross-sectional studies, offering only a snapshot of companion animals’ impacts (Batty et al., 2017). Moreover, the complexity and heterogeneity of findings could also be due to regional (Enmarker et al., 2015), cultural, and sample variabilities (Hajek and König, 2020). Additionally, the mental health effects may differ based on the type of companion animals and the specific traits of the companion animals themselves. Therapy animals, known for their friendliness, obedience, and approachability, may interact more effectively with humans, enhancing mental health. This may be where the differences lie in how various types of companion animals influence mental health (Bradley and Bennett, 2015). Importantly, although uncontrolled variables indicate companion animals might positively affect children’s health and development, controlling these variables often nullifies the evidence for such benefits (Miles et al., 2017). Nonetheless, certain scholars argue that the absence of significant findings could stem from inadequate group influence, sample selection biases, and homogeneity concerns (Miller and Lago, 1990). This may also be attributed to evaluation consistency (close relationships between subjects and their companion animals may lead to overly positive assessments, potentially masking individual differences in companion animals’ actual impact on mental health), complex dynamics during therapy (additional psychological distress may arise from challenges related to companion animals care and concerns about the companion animals’ future), limitations of assessment tools, variability in companion animals traits, and individual preferences (Ingram and Cohen-Filipic, 2019).

### 4.2 Negative impacts of companion animals

Besides the studies yielding inconclusive results, research suggests that companion animals can adversely affect human psychological health. For example, Bradley and Bennett (2015) and Ingram and Cohen-Filipic (2019) found that companion animals caregiving could induce depression, stress, and anxiety. This could stem from the substantial time, energy, and finances needed for companion animals care (Ingram and Cohen-Filipic, 2019), especially for individuals preoccupied with work or financially constrained, further intensifying stress (Christiansen et al., 2013; Goldberg, 2017; Spitznagel et al., 2017). Conversely, it might be that individuals adopt companion animals due to depression rather than companion animals ownership causing it (Liu et al., 2019). Batty et al. (2017) suggested that companion animals ownership might intensify owners’ loneliness. This could be due to decreased social interaction among owners, as having a companion animal might cause them to engage less with people and more with their companion animals, heightening loneliness. Wong et al. (2017) and Rémillard et al. (2017) noted that companion animal loss or health problems can provoke grief and negative emotions in owners, similar to losing a close person, and may lead to depression, guilt, and psychological distress. These emotional reactions can profoundly impact an individual’s mental well-being and life quality. Messam and Hart (2019) even discovered that, in some instances, these effects could linger for years.

## 5 The impact of companion animals on specific demographics

In examining the impact of companion animals on human psychological health, it’s crucial to acknowledge that this influence may differ across diverse demographic groups. These variances are manifested not only in individual psychological and physiological traits, like animal allergies, fear responses, and gender, but also in social factors, including marital status and interpersonal relations.

Individuals prone to allergies may find companion animals a substantial stressor. Wells (2009) noted that companion animals could intensify allergic individuals’ stress responses and elevate certain disease risks, including asthma (Gergen et al., 2018) and zoonotic diseases (Damborg et al., 2016). Such physiological adversities might also impair mental health.

Gulick and Krause-Parello (2012) observed that cat-owning women report heightened depression and intensified loneliness. Tower and Nokota (2006) stated that pet-owning unmarried women exhibit the least depression, in contrast to unmarried men. These disparities could stem from gender roles and societal expectations: women often derive emotional satisfaction from companion animal care, whereas men might react differently due to distinct societal roles. Unmarried men might view companion animal ownership as stressful, demanding additional effort and attention, possibly impinging on their independence and autonomy.

Clearly, companion animals’ effects on mental health are not uniform but influenced by multifaceted factors. The potential pathways to human health enhancement are intricate, precluding any simplistic classification of companion animals’ impact as purely positive or negative. Moreover, it’s important to note that the link between animals and human health is correlational rather than causal. Thus, companion animal ownership should not be oversimplified as a method to enhance human health. Concurrently, recognizing companion animals’ dual role in mental health is crucial: they can support psychological well-being, but their potential adverse effects must not be overlooked.

## 6 Attachment

Attachment theory, proposed by John Bowlby, seeks to clarify the deep emotional connections between humans and their caregivers in early development. Recently, the scope of this theory has broadened to include human-companion animal relationships. Research indicates that humans and companion animals exhibit comparable attachment patterns, both behaviorally and physiologically (Zilcha-Mano et al., 2011; Solomon et al., 2019), primarily evidenced by care, protection, and emotional investment (Archer, 1997). Research has shown that emotional bonds with companion animals profoundly affect human psychological health (Wells, 2019). For instance, higher companion animal attachment scores correlate with improved executive functioning (Branson et al., 2016). One rationale is that active interaction with animals in their presence enriches the stimuli individuals receive. Companion animals offer positive social engagement, psychological solace, and physical activity, thus ameliorating stress responses and enhancing executive functions. However, the causality of this outcome is unclear: it’s uncertain whether companion animal interaction promotes higher activity levels and thus better executive functions, or if individuals with robust executive functions are more inclined to own companion animals. Oxytocin, a hormone produced in the hypothalamus and released during childbirth and lactation, plays a role in various physiological and psychological processes like stress management, social interactions, and pair bonding (Feldman et al., 2016). Studies indicate that the close bond between companion animal owners and their companion animals might enhance the owner’s psychological health by stimulating the oxytocin system (Wells, 2019).

The link between companion animal attachment and psychological health is not solely beneficial. Studies indicate that overly attached individuals may face heightened psychological distress (Peacock et al., 2012). This could stem from over-dependence on companion animals for emotional support, triggering adverse psychological responses upon companion animal loss or separation. Antonacopoulos and Pychyl (2010) observed that companion animal owners with scant social support and strong companion animal attachment report elevated loneliness and depression scores. Excessive reliance on companion animals might result in neglecting human social connections. Consequently, it’s uncertain whether strong companion animal attachment induces depression and loneliness, or if individuals predisposed to these feelings are more likely to form close bonds with companion animals. Ingram and Cohen-Filipic (2019) suggested that patients in ongoing treatment with strong companion animal attachments might have worsened psychological health. This may result from the treatment-induced physical limitations, complicating companion animal care activities like dog walking and heightening concerns about companion animal welfare post-owner demise.

Furthermore, attachment relationships vary between humans and different companion animal species. Endo et al. (2020) found a correlation between companion animal ownership and children’s well-being: 10-year-olds with dogs were happier at 12, in contrast to their peers with cats. This could be attributed to the increased physical activity, social engagement, and stronger emotional bonds provided by dogs, whereas cat ownership may entail physiological and psychological risks like toxoplasmosis. A dog’s gaze may elevate oxytocin levels in the owner (Nagasawa et al., 2009), fostering social ties, reducing stress, and enhancing psychological well-being. However, such methodologies have not been used in cat studies, leaving the effect of cat ownership on oxytocin levels unclear, and warranting additional research.

The bond between companion animals and humans is multifaceted: while companion animal attachment usually benefits human psychological health, it may yield adverse effects, particularly when it becomes excessive or detrimental. Consequently, discerning and distinguishing between healthy and unhealthy companion animal attachment is essential to maximize the role of companion animals in improving psychological well-being.

## 7 Animal-assisted interventions

Animal-assisted interventions include Animal-Assisted Therapy (AAT), Animal-Assisted Activities (AAA), and Animal-Assisted Education/Learning (AAE/AAL). These interventions involve specially trained animals and are administered by skilled mental health professionals to provide therapeutic, educational, or recreational benefits, aiming to enhance physical, social, emotional, and cognitive functions in humans (O’Callaghan, 2008). Documented first in the 1960s by mental health professionals, Levinson observed that a withdrawn child patient began interacting after encountering Levinson’s dog (Mallon, 1999). It has been reported that a single session of animal-assisted therapy can be as effective as ten traditional psychotherapy sessions (O’Callaghan, 2008). This effectiveness is largely due to the animal’s presence significantly reducing visitors’ defensive mechanisms, especially in those with resistant emotional states. This facilitates therapists in engaging more effectively with clients and advancing the therapeutic process. Currently, animal-assisted interventions are widely used for various psychological conditions, including depression (Souter and Miller, 2007; Berget et al., 2011; Berget and Braastad, 2011), anxiety disorders (Barker and Dawson, 1998; Nimer and Lundahl, 2007), and autism spectrum disorders (O’Haire, 2013; O’Haire et al., 2013), with the latter being one of the most responsive areas to this intervention (Zhou et al., 2019).

Nimer and Lundahl (2007) utilized meta-analysis to explore the effects of animal-assisted interventions, finding moderate effectiveness in areas such as autism spectrum disorders, medical challenges, behavioral issues, and emotional health. Subgroup analyzes indicated that therapy dogs consistently demonstrated the most effective outcomes, maintaining high efficacy across various studies. However, the impact of horses, aquatic animals, and other animals showed more variability. Despite the use of various animals in contemporary interventions, including horses (Tseng et al., 2013; Collado-Mateo et al., 2020; Prieto et al., 2022), dolphins (Dilts, 2008; Matamoros et al., 2022), cats (Goleman et al., 2012), and birds (Colombo et al., 2006), therapy dogs have emerged as the most widely used, historically established, and effective therapeutic animals (Chandler, 2001).

Findings indicate that children, compared to other age groups, benefit more substantially from different therapeutic approaches. This phenomenon may be attributed to the “biophilia” concept, which suggests an inherent love for life and living systems (Wells, 2019). Wilson (cited in Wells, 2019) argues that humans possess an innate ability to perceive animals and a tendency to focus attention on them, particularly evident in early life stages. Research shows that infants tend to pay more attention to animals than inanimate objects (DeLoache et al., 2011). This natural inclination likely enhances the attention child patients give to animals, thereby potentially increasing the effectiveness of animal-assisted interventions for this demographic.

However, some meta-analyzes have found that animal-assisted interventions produce positive effects in a limited number of cases (Feng et al., 2021). These inconsistencies may arise due to factors such as small sample sizes, lack of control groups, sample bias, and short durations of interventions (Ma et al., 2019; Liu and Gao, 2021). Therefore, further research employing more comprehensive experimental designs is needed to elucidate the regulatory mechanisms behind these disparate outcomes in animal-assisted interventions.

Although animal-assisted interventions have achieved significant success in treating various psychological issues and are known for their minimal side effects and overall effectiveness (Liu and Gao, 2021). An analysis of 18 studies revealed that despite the limited efficacy of animal-assisted interventions in special education, no adverse impacts were reported (Meixner and Kotrschal, 2022). Nonetheless, animal-assisted interventions are nascent globally, with notable deficits in their application and research in certain countries, such as China (Ma et al., 2019). The relationship between humans and animals, which varies significantly across different cultural backgrounds (Pagani et al., 2007; Zalaf and Egan, 2017; Randler et al., 2021), is influenced by various factors such as attitudes towards animals and levels of attachment (Gulick and Krause-Parello, 2012). Consequently, the effects of animal-assisted interventions in different national contexts are largely unknown, highlighting the need for further development in research and application tailored to diverse socio-cultural backgrounds.

## 8 Neural basis of companion animals’ impact on human emotions

Many studies highlight companion animals’ positive effects on human psychological health, yet questionnaires fall short in clarifying causality, and conducting stringent longitudinal experiments proves difficult. Investigating cognitive neural activity changes during human-companion animal interactions may more accurately reveal companion animals’ potential impact on psychological health. Hence, this section intends to compile and synthesize research on companion animals’ influence on human neural activities, deducing neural mechanisms from existing literature. This provides a cognitive-neural basis for comprehending how companion animals improve psychological health, and supports domains such as animal-assisted intervention.

### 8.1 Frontal cortex

In studies examining the influence of companion animals on human neural activity, the frontal cortex is one of the most frequently mentioned brain regions. Kobayashi et al. (2017) used functional near-infrared spectroscopy (fNIRS) to compare brain neural activity when participants stroked a real versus a toy cat, finding that stroking a real cat led to higher activation levels in the inferior frontal gyrus and more positive emotions. Functional magnetic resonance imaging studies have shown that viewing companion animal photos significantly enhances activity in the frontal cortex areas. Moreover, companion animal owners exhibit greater activation in areas like the insular cortex compared to non-companion animal owners (Hayama et al., 2016). Additionally, neural activity in other frontal cortex regions, such as the left middle frontal gyrus (MFG) and the right frontopolar area, is also influenced by companion animals, coinciding with emotional changes (Sugawara et al., 2012; Matsuura et al., 2020).

The frontal lobe, vital for cognitive control and emotional regulation, assists in modulating emotional responses (Etkin et al., 2011). Emotional disorders’ emergence and persistence are linked to the frontal lobe; its functional anomalies can disrupt emotional regulation (Kohn et al., 2014; Pressman and Rosen, 2015), affecting psychological health. Studies show notable differences in frontal lobe activation between depressed patients and healthy individuals, mainly marked by reduced frontal cortex activity (Herrmann et al., 2004; Liu et al., 2016). This correlates with depressive symptoms like emotional regulation disturbances (Marusak et al., 2015), executive function impairments (Carpenter et al., 2000), and working and episodic memory deficits (Balconi, 2013). Current research indicates that neuro-modulation techniques like Transcranial Magnetic Stimulation (TMS) and Transcranial Direct Current Stimulation (tDCS) can modify frontal lobe neural activity, alleviating depression symptoms (Herrmann and Ebmeier, 2006; Boggio et al., 2008). The frontal cortex can regulate amygdala activity, thus influencing the intensity and duration of emotional reactions. In individuals with emotional disorders, diminished frontal lobe function reduces the ability to manage emotional responses, resulting in emotional abnormalities (Shin and Liberzon, 2010). Consequently, interactions with companion animals may strengthen the human cognitive control system and enhance social interactions. Frontal lobe activation could be a neural mechanism through which companion animals positively influence psychological health, as companion animal engagement may bolster the frontal lobe’s emotional regulation capabilities, thereby improving emotions.

### 8.2 Amygdala

Stoeckel et al. (2014) discovered that mothers experienced a significant increase in amygdala activity when viewing photos of their children and pet dogs. The researchers suggest that the amygdala, a key brain area in forming intimate relationships, generates emotional and motivational responses while processing these images, vital for developing close bonds. Additionally, a study by Mormann et al. (2011) comparing neural activities in response to animal versus non-animal images found that animal pictures activated the right amygdala independently of valence and arousal. Mormann and colleagues theorize that the right amygdala’s specialized function in processing animal information may have evolutionary significance, indicating that the amygdala’s role in processing companion animal information could extend beyond emotional processing to specialized cognitive processing of animal information.

The amygdala is vital for emotion processing, particularly in handling negative emotions like fear, anger, and anxiety (Janak and Tye, 2015). Amygdala abnormalities can disrupt neural circuits for emotional regulation, potentially causing excessive or insufficient emotional responses in those with emotional disorders (Drevets, 2003). For example, in anxiety disorder patients, heightened amygdala activation to emotional faces (anger, fear, happiness) exceeds that in healthy individuals, complicating the suppression of excessive emotional responses (Shin and Liberzon, 2010). The reason might be that these emotional responses in patients do not subside. When a stimulus previously associated with anger or fear no longer carries dangerous information, patients cannot rapidly learn or adapt to this change, resulting in their sustained fearful and anxious responses to past stimuli (Shin and Liberzon, 2010). Beesdo et al. (2009) noted that when faced with fear stimuli, adolescent patients with depression and anxiety disorders showed heightened amygdala activation compared to healthy controls. Recent findings indicate that the amygdala reacts to positive and neutral emotions like music, not solely to negative stimuli (Armony, 2013). Sander et al. (2003) propose that the amygdala responds not just to conventional threats but also to novel or unexpected stimuli with potential biological relevance (Sander et al., 2003). As non-threatening biological stimuli, companion animals’ presence in human lives may be recognized by the brain as a biologically significant novel event. This novelty may activate the amygdala, initiating a cascade of emotional and cognitive reactions. Consequently, companion animals might mitigate excessive or abnormal emotional reactions by influencing the amygdala’s processing of emotional information.

### 8.3 Autonomic nervous system

The autonomic nervous system, divided into the sympathetic and parasympathetic nervous systems, is distributed across the internal organs, cardiovascular system, and glands, regulating physiological activities like heartbeat, respiration, and digestion. Studies have documented significant decreases in heart rate and blood pressure in humans following interactions with companion animals (Allen et al., 2002; Odendaal and Meintjes, 2003; Polheber and Matchock, 2014). For instance, Polheber and Matchock (2014) found that subjects performing stress tasks in the presence of companion animal dogs exhibited significantly lower heart rates than those accompanied by friends or in control groups. This suggests that companion animals may modulate autonomic nervous system activity by providing social support that reduces stress. Unlike the social support from friends, which may induce evaluation anxiety, the presence of pet dogs does not have this negative effect, thereby offering more effective emotional regulation. Conversely, a study showed increased arousal levels in participants after interacting with pet cats, attributed to a positive psychophysiological response from benign stress, which enhances vitality and promotes health (Nagasawa et al., 2023).

The sympathetic nervous system plays a key role in stress responses, such as increasing heart rate, blood pressure, and triggering responses to emergencies. It activates in stressful or dangerous situations, aiding survival and adaptation. However, prolonged stress responses can have detrimental physiological effects, like impaired immune function, increased cardiovascular disease risk, and endocrine disturbances (Chrousos, 2009). Excessive stress can also negatively impact mental health, leading to emotional disorders, insomnia, and irritability (Yaribeygi et al., 2017). The parasympathetic nervous system, on the other hand, can lower heart rate and blood pressure, preventing damage from excessive stress responses. The interaction between these two systems maintains homeostasis. Therefore, the presence of companion animals may buffer the adverse effects of stress by regulating autonomic nervous system activity.

### 8.4 Oxytocin levels

Oxytocin, as a neurohormone, plays an essential role in social behavior and emotional states (Feldman et al., 2016), and interaction with companion animals can modulate oxytocin secretion in humans. Nagasawa et al. (2009) found that oxytocin levels in the urine of pet dog owners significantly increased when their dogs gazed at them. Odendaal and Meintjes (2003) noted a rise in oxytocin levels in both pet dogs and their owners during interactions, correlating with the intimacy of their relationship. Recent research also suggests that interactions with household cats may influence the human oxytocin system (Nagasawa et al., 2023).

Oxytocin is vital for trust, intimate bonds, and emotional regulation. Oxytocin also mitigates negative emotions like anxiety, depression, and stress. Heinrichs et al. (2003) found that intranasal oxytocin lowers cortisol levels during psychological stress, thus soothing individuals. Cortisol, a recognized stress biomarker (Russell et al., 2012), suggests that oxytocin could affect psychological health by modulating other hormone levels. Moreover, oxytocin enhances interpersonal interactions, social bonds (MacDonald and MacDonald, 2010), and provides physiological advantages, including bolstered immune and cardiovascular functions (Creagan et al., 2015). During human-companion animal interactions, companion animals can stimulate human oxytocin release, fostering pleasure and relaxation, and reinforcing trust and intimacy (Beetz et al., 2012; Marcus, 2013).

### 8.5 Cortisol levels

Cortisol, a steroid hormone produced by the adrenal cortex, plays a role in stress response, glucose metabolism, and immune function (Tsigos and Chrousos, 2002). Interactions with companion animals can also regulate human cortisol levels. For instance, oxytocin levels significantly rise while cortisol levels decrease in both owners and their pet dogs during interactions (Handlin et al., 2011). Similar results have been observed in unique settings such as hospitals. Beetz et al. (2011) found that cortisol levels in children with insecure attachment decreased after interacting with pet dogs, unlike interactions with friendly humans or toy dogs. Therapy dogs have also been proven to reduce cortisol levels in caregivers, lower stress levels, and enhance immunity (Barker et al., 2005; Barker and Wolen, 2008).

Cortisol mirrors the hypothalamic–pituitary–adrenal (HPA) axis’s activity. The HPA axis specifically responds to psychological stress, with cortisol concentration changes effectively indicating stress levels (Nater et al., 2013; Vives et al., 2015). However, sustained high cortisol levels may adversely affect both physical and psychological health. For example, it can precipitate emotional problems (Staufenbiel et al., 2013), impair cognitive functions (Ouanes and Popp, 2019), and trigger significant physical health issues (Schoorlemmer et al., 2009; Pivonello et al., 2016). Human-companion animal interactions can effectively mitigate stress responses and decrease cortisol levels during stressful periods.

### 8.6 Other neurohormonal levels

In addition to oxytocin and cortisol, interactions with companion animals can also influence the secretion of other emotion-related hormones, such as serotonin, dopamine, phenylethylamine, endorphins, and prolactin (Odendaal and Meintjes, 2003; Creagan et al., 2015). For example, human interaction with pet dogs has been shown to increase the release of phenylethylamine, leading to heightened positive emotions and sociability. Notably, individuals without companion animals exhibit higher phenylethylamine release during interactions with dogs compared to companion animal owners (Odendaal and Lehmann, 2000). Involving pet dogs in the care of children and adolescents with cancer has been observed to change neurotransmitter levels, producing analgesic effects and achieving clinically meaningful pain relief (Harper et al., 2015). Typically, interactions with companion animals result in changes in the secretion of multiple neurohormones, which collectively regulate changes in human emotions.

## 9 Limitations and prospects

Although current research underscores the significant impact of companion animals on human psychological health and neural activities, this field of study is still in its early stages and inevitably has some limitations.

Current research often employs resting-state methods to explore the effects of companion animals on human neural activity. This approach compares neural activity differences between human-companion animal interaction and control groups, or before and after interactions, to illustrate the effects. However, this method primarily reveals the short-term impact of brief human-companion animal interactions on neural activity and does not reflect the enduring effects of long-term companionship with companion animals. Future research should utilize longitudinal experimental designs to investigate the long-term effects of prolonged human-companion animal interactions on psychological health. Moreover, studies should explore the impact of companion animals on various aspects of emotional cognitive processing, such as emotion perception, attention, and memory. Observing changes in emotional cognitive processing could help infer potential long-lasting changes in psychological health.

An increasing number of scholars argue that cognitive function relies not just on isolated brain regions but on the communication between different areas of the brain (Axer and Amunts, 2022; Lee et al., 2022; Thiebaut de Schotten and Forkel, 2022). However, much current research has focused on the activation of individual brain regions. Studies examining companion animal images have found that companion animal-related information activates specific neural networks (Kojima et al., 2013; Cao et al., 2014; Fang et al., 2016), potentially triggering brain network activities associated with rewards, emotions, belonging, and animal-specific responses (Kojima et al., 2013; Stoeckel et al., 2014). Future research should incorporate functional connectivity analysis to explore companion animal-induced brain network activities, viewing companion animals’ impact on human neural activities through the lens of brain connectivity.

As previously mentioned, companion animals may serve as “friends” or “family members” providing social support to humans (Teo and Thomas, 2019), and triggering activity in related human brain regions (Stoeckel et al., 2014), thereby regulating emotions and enhancing mental health, a characteristic lacking in many positive stimuli. Although the specificity of companion animal-related information has been proven by research, it is still unknown whether its effect on human emotions and mental health differs from that of ordinary positive stimuli. Therefore, future studies could explore the similarities and differences in the effects of companion animals and other positive stimuli on human emotions and mental health from a cognitive neuroscience perspective, providing guidance for related applied research.

Research indicates that indirect interactions with companion animals, such as watching companion animal videos (Wells, 2005; Myrick, 2015; Ein et al., 2023) and pictures (Ein et al., 2019), can regulate emotions. However, there are fewer studies on the long-term psychological impacts of indirect companion animal contact. In modern society, “virtual companion animal raising” has become a substantial trend, where individuals unable to own companion animals satisfy their psychological needs through online companion animal-related content (Nie, 2020). Studies suggest that virtual companion animal raising might positively impact human psychological health (Myrick, 2015), but due to limited research, the stability of this effect and its difference from direct companion animal contact remain unclear. Future research should investigate the psychological impact of virtual companion animal raising and its cognitive and neural mechanisms.

Numerous studies have found that different types of companion animals have varying impacts on human psychology (Hajek and König, 2020; Oliva and Johnston, 2021; Matsumura et al., 2022), leading researchers to focus on these differences. However, current research mainly compares companion animal ownership with non-ownership and often overlooks companion animals other than cats and dogs (Fraser et al., 2020). Consequently, investigating the differential impacts of various types of pets, particularly unconventional ones such as spiders and snakes, on mental health can better elucidate the underlying cognitive mechanisms and also facilitate the development of related applications.

Attitudes towards animals and companion animals significantly vary across countries (Zalaf and Egan, 2015; Martens et al., 2019; Randler et al., 2021) and cultural backgrounds (Pagani et al., 2007; Zalaf and Egan, 2017). These attitudes and the nature of human-companion animal relationships significantly modulate companion animals’ impact on humans (Headey et al., 2008; Fraser et al., 2020). With most research data in this field coming from Western countries, there is a need for more studies in diverse populations to understand potential differences (Randler et al., 2021).

## Author contributions

HL: Data curation, Software, Visualization, Writing – original draft, Writing – review & editing. JL: Conceptualization, Investigation, Resources, Supervision, Writing – review & editing. WL: Conceptualization, Funding acquisition, Investigation, Methodology, Supervision, Writing – review & editing.

## References

[ref1] Allen K. Blascovich J. Mendes W. B. (2002). Cardiovascular reactivity and the presence of pets, friends, and spouses: the truth about cats and dogs. Psychosom. Med. 64, 727–739. doi: 10.1097/01.PSY.0000024236.11538.41, PMID: 12271103

[ref2] Anderson A. P. Mayer M. D. Fellows A. M. Cowan D. R. Hegel M. T. Buckey J. C. (2017). Relaxation with immersive natural scenes presented using virtual reality. Aerosp. Med. Hum. Perform. 88, 520–526. doi: 10.3357/AMHP.4747.2017 28539139

[ref3] Antonacopoulos N. M. D. Pychyl T. A. (2010). An examination of the potential role of pet ownership, human social support and pet attachment in the psychological health of individuals living alone. Anthrozoös 23, 37–54. doi: 10.2752/175303710X12627079939143

[ref4] Archer J. (1997). Why do people love their pets? Evol. Hum. Behav. 18, 237–259. doi: 10.1016/S0162-3095(99)80001-4

[ref5] Armony J. L. (2013). Current emotion research in behavioral neuroscience: the role (s) of the amygdala. Emot. Rev. 5, 104–115. doi: 10.1177/1754073912457208

[ref6] Axer M. Amunts K. (2022). Scale matters: the nested human connectome. Science 378, 500–504. doi: 10.1126/science.abq2599, PMID: 36378967

[ref7] Azevedo A. Guimarães L. Ferraz J. Whiting M. Magalhães-Santana M. (2022). Understanding the human–reptile bond: an exploratory mixed-methods study. Anthrozoös 35, 755–772. doi: 10.1080/08927936.2022.2051934

[ref8] Bachi K. Parish-Plass N. (2017). Animal-assisted psychotherapy: a unique relational therapy for children and adolescents. Clin. Child Psychol. Psychiatry 22, 3–8. doi: 10.1177/1359104516672549, PMID: 27742758

[ref9] Balconi M. (2013). Dorsolateral prefrontal cortex, working memory and episodic memory processes: insight through transcranial magnetic stimulation techniques. Neurosci. Bull. 29, 381–389. doi: 10.1007/s12264-013-1309-z, PMID: 23385388 PMC5561838

[ref10] Bao K. J. Schreer G. (2016). Pets and happiness: examining the association between pet ownership and wellbeing. Anthrozoös 29, 283–296. doi: 10.1080/08927936.2016.1152721

[ref11] Barker S. B. Dawson K. S. (1998). The effects of animal-assisted therapy on anxiety ratings of hospitalized psychiatric patients. Psychiatr. Serv. 49, 797–801. doi: 10.1176/ps.49.6.797, PMID: 9634160

[ref12] Barker S. B. Knisely J. S. McCain N. L. Best A. M. (2005). Measuring stress and immune response in healthcare professionals following interaction with a therapy dog: a pilot study. Psychol. Rep. 96, 713–729. doi: 10.2466/pr0.96.3.713-729 16050629

[ref13] Barker S. B. Wolen A. R. (2008). The benefits of human–companion animal interaction: a review. J. Vet. Med. Educ. 35, 487–495. doi: 10.3138/jvme.35.4.487, PMID: 19228898

[ref14] Barklam E. B. Felisberti F. M. (2023). Pet ownership and wellbeing during the COVID-19 pandemic: the importance of resilience and attachment to pets. Anthrozoös 36, 215–236. doi: 10.1080/08927936.2022.2101248

[ref15] Batty G. D. Zaninotto P. Watt R. G. Bell S. (2017). Associations of pet ownership with biomarkers of ageing: population based cohort study. BMJ 359:j5558. doi: 10.1136/bmj.j5558, PMID: 29237607 PMC5728306

[ref16] Beesdo K. Lau J. Y. Guyer A. E. McClure-Tone E. B. Monk C. S. Nelson E. E.. (2009). Common and distinct amygdala-function perturbations in depressed vs anxious adolescents. Arch. Gen. Psychiatry 66, 275–285. doi: 10.1001/archgenpsychiatry.2008.545, PMID: 19255377 PMC2891508

[ref17] Beetz A. M. (2017). Theories and possible processes of action in animal assisted interventions. Appl. Dev. Sci. 21, 139–149. doi: 10.1080/10888691.2016.1262263

[ref18] Beetz A. Kotrschal K. Turner D. C. Hediger K. Uvnäs-Moberg K. Julius H. (2011). The effect of a real dog, toy dog and friendly person on insecurely attached children during a stressful task: an exploratory study. Anthrozoös 24, 349–368. doi: 10.2752/175303711X13159027359746

[ref19] Beetz A. Uvnäs-Moberg K. Julius H. Kotrschal K. (2012). Psychosocial and psychophysiological effects of human-animal interactions: the possible role of oxytocin. Front. Psychol. 3:234. doi: 10.3389/fpsyg.2012.00234, PMID: 22866043 PMC3408111

[ref21] Berget B. Braastad B. O. (2011). Animal-assisted therapy with farm animals for persons with psychiatric disorders. Ann. Ist. Super. Sanita. 47, 384–390. doi: 10.4415/ANN_11_04_10 22194073

[ref22] Berget B. Ekeberg Ø. Pedersen I. Braastad B. O. (2011). Animal-assisted therapy with farm animals for persons with psychiatric disorders: effects on anxiety and depression, a randomized controlled trial. Occup. Ther. Ment. Health 27, 50–64. doi: 10.1080/0164212X.2011.543641

[ref24] Boggio P. S. Rigonatti S. P. Ribeiro R. B. Myczkowski M. L. Nitsche M. A. Pascual-Leone A.. (2008). A randomized, double-blind clinical trial on the efficacy of cortical direct current stimulation for the treatment of major depression. Int. J. Neuropsychopharmacol. 11, 249–254. doi: 10.1017/S1461145707007833, PMID: 17559710 PMC3372849

[ref25] Borgi M. Cirulli F. (2016). Pet face: mechanisms underlying human-animal relationships. Front. Psychol. 7:298. doi: 10.3389/fpsyg.2016.00298, PMID: 27014120 PMC4782005

[ref26] Bradley L. Bennett P. C. (2015). Companion-animals’ effectiveness in managing chronic pain in adult community members. Anthrozoös 28, 635–647. doi: 10.1080/08927936.2015.1070006

[ref27] Branson S. Boss L. Cron S. Kang D. H. (2016). Examining differences between homebound older adult pet owners and non-pet owners in depression, systemic inflammation, and executive function. Anthrozoös 29, 323–334. doi: 10.1080/08927936.2016.1152764

[ref29] Brooks H. L. Rushton K. Lovell K. Bee P. Walker L. Grant L.. (2018). The power of support from companion animals for people living with mental health problems: a systematic review and narrative synthesis of the evidence. BMC Psychiatry 18, 31–12. doi: 10.1186/s12888-018-1613-2, PMID: 29402247 PMC5800290

[ref30] Cao Z. Zhao Y. Tan T. Chen G. Ning X. Zhan L.. (2014). Distinct brain activity in processing negative pictures of animals and objects—the role of human contexts. NeuroImage 84, 901–910. doi: 10.1016/j.neuroimage.2013.09.064, PMID: 24099847 PMC3849327

[ref31] Carpenter P. A. Just M. A. Reichle E. D. (2000). Working memory and executive function: evidence from neuroimaging. Curr. Opin. Neurobiol. 10, 195–199. doi: 10.1016/S0959-4388(00)00074-X 10753796

[ref32] Cevizci S. Babaoglu U. T. Erginiöz E. Işsever H. (2012). The relation of pet ownership, psychological stress, regular physical exercise and smoking in white-collar workers of a special company in beşiktaş region of Istanbul. Nobel Medicus 53, 52–59. doi: 10.1007/978-3-319-01285-8_6

[ref33] Chandler C. (2001). Animal assisted therapy in counseling. ERIC counseling and student services clearinghouse, University of North Carolina at Greensboro. Available at: http://ericcass

[ref34] Chang S. J. Lee J. An H. Hong W. H. Lee J. Y. (2021). Animal-assisted therapy as an intervention for older adults: a systematic review and meta-analysis to guide evidence-based practice. Worldviews Evid.-Based Nurs. 18, 60–67. doi: 10.1111/wvn.12484, PMID: 33277977

[ref35] Cheung C. K. Kam P. K. (2018). Conditions for pets to prevent depression in older adults. Aging Ment. Health 22, 1627–1633. doi: 10.1080/13607863.2017.1385723, PMID: 28976782

[ref36] Christiansen S. B. Kristensen A. T. Sandøe P. Lassen J. (2013). Looking after chronically iii dogs: impacts on the caregiver’s life. Anthrozoös 26, 519–533. doi: 10.2752/175303713X13795775536174

[ref37] Chrousos G. P. (2009). Stress and disorders of the stress system. Nat. Rev. Endocrinol. 5, 374–381. doi: 10.1038/nrendo.2009.1060 19488073

[ref39] Clements H. Valentin S. Jenkins N. Rankin J. Baker J. S. Gee N.. (2019). The effects of interacting with fish in aquariums on human health and well-being: a systematic review. PLoS One 14:e0220524. doi: 10.1371/journal.pone.0220524 31356652 PMC6663029

[ref40] Collado-Mateo D. Lavín-Pérez A. M. Fuentes García J. P. García-Gordillo M. Á. Villafaina S. (2020). Effects of equine-assisted therapies or horse-riding simulators on chronic pain: a systematic review and meta-analysis. Medicina 56:444. doi: 10.3390/medicina56090444, PMID: 32878327 PMC7557603

[ref41] Colombo G. Buono M. D. Smania K. Raviola R. De Leo D. (2006). Pet therapy and institutionalized elderly: a study on 144 cognitively unimpaired subjects. Arch. Gerontol. Geriatr. 42, 207–216. doi: 10.1016/j.archger.2005.06.011 16191447

[ref42] Creagan E. T. Bauer B. A. Thomley B. S. Borg J. M. (2015). Animal-assisted therapy at Mayo Clinic: the time is now. Complement. Ther. Clin. Pract. 21, 101–104. doi: 10.1016/j.ctcp.2015.03.002 25900612

[ref43] Cui Y. Russell M. Davern M. Christian H. (2021). Longitudinal evidence of the impact of dog ownership and dog walking on mental health. J. Public Health 43, e145–e152. doi: 10.1093/pubmed/fdz094 31690938

[ref44] Curl A. L. Bibbo J. Johnson R. A. (2021). Neighborhood engagement, dogs, and life satisfaction in older adulthood. J. Appl. Gerontol. 40, 1706–1714. doi: 10.1177/0733464820953725, PMID: 32909494

[ref45] Damborg P. Broens E. M. Chomel B. B. Guenther S. Pasmans F. Wagenaar J. A.. (2016). Bacterial zoonoses transmitted by household pets: state-of-the-art and future perspectives for targeted research and policy actions. J. Comp. Pathol. 155, S27–S40. doi: 10.1016/j.jcpa.2015.03.004, PMID: 25958184

[ref46] DeLoache J. S. Pickard M. B. LoBue V. (2011). “How very young children think about animals” in How animals affect us: Examining the influences of human–animal interaction on child development and human health. eds. McCardle P. McCune S. Griffin J. A. Maholmes V. (Washington, DC: American Psychological Association), 85–99.

[ref47] DeSchriver M. M. Riddick C. C. (1990). Effects of watching aquariums on elders’ stress. Anthrozoös 4, 44–48. doi: 10.2752/089279391787057396

[ref48] Dilts R. M. (2008). A summative evaluation of a dolphin assisted therapy program for children with special needs Oregon State University. Corvallis, OR

[ref49] Dimitrijević I. (2009). Animal-assisted therapy–a new trend in the treatment of children and adults. Psychiatr. Danub. 21, 236–241. PMID: 19556955

[ref50] Drevets W. C. (2003). Neuroimaging abnormalities in the amygdala in mood disorders. Ann. N. Y. Acad. Sci. 985, 420–444. doi: 10.1111/j.1749-6632.2003.tb07098.x 12724175

[ref51] Ein N. Gervasio J. Reed M. J. Vickers K. (2023). Effects on wellbeing of exposure to dog videos before a stressor. Anthrozoös 36, 349–367. doi: 10.1080/08927936.2022.2149925

[ref52] Ein N. Hadad M. Reed M. J. Vickers K. (2019). Does viewing a picture of a pet during a mental arithmetic task lower stress levels? Anthrozoös 32, 519–532. doi: 10.1080/08927936.2019.1621524

[ref53] Endenburg N. van Lith H. A. (2011). The influence of animals on the development of children. Vet. J. 190, 208–214. doi: 10.1016/j.tvjl.2010.11.020 21195645

[ref54] Endo K. Yamasaki S. Ando S. Kikusui T. Mogi K. Nagasawa M.. (2020). Dog and cat ownership predicts adolescents’ mental well-being: a population-based longitudinal study. Int. J. Environ. Res. Public Health 17:884. doi: 10.3390/ijerph17030884, PMID: 32023841 PMC7037461

[ref55] Enmarker I. Hellzén O. Ekker K. Berg A. G. T. (2015). Depression in older cat and dog owners: the Nord-Trøndelag health study (HUNT)-3. Aging Ment. Health 19, 347–352. doi: 10.1080/13607863.2014.933310 24990174

[ref56] Etkin A. Egner T. Kalisch R. (2011). Emotional processing in anterior cingulate and medial prefrontal cortex. Trends Cogn. Sci. 15, 85–93. doi: 10.1016/j.tics.2010.11.004, PMID: 21167765 PMC3035157

[ref57] Fang Z. Li H. Chen G. Yang J. (2016). Unconscious processing of negative animals and objects: role of the amygdala revealed by fMRI. Front. Hum. Neurosci. 10:146. doi: 10.3389/fnhum.2016.00146, PMID: 27092067 PMC4820445

[ref58] Feldman R. Monakhov M. Pratt M. Ebstein R. P. (2016). Oxytocin pathway genes: evolutionary ancient system impacting on human affiliation, sociality, and psychopathology. Biol. Psychiatry 79, 174–184. doi: 10.1016/j.biopsych.2015.08.008, PMID: 26392129

[ref59] Feng Y. Lin Y. Zhang N. Jiang X. Zhang L. (2021). Effects of animal-assisted therapy on hospitalized children and teenagers: a systematic review and meta-analysis. J. Pediatr. Nurs. 60, 11–23. doi: 10.1016/j.pedn.2021.01.020, PMID: 33582447

[ref60] Fraser G. Huang Y. Robinson K. Wilson M. S. Bulbulia J. Sibley C. G. (2020). New Zealand pet owners’ demographic characteristics, personality, and health and wellbeing: more than just a fluff piece. Anthrozoös 33, 561–578. doi: 10.1080/08927936.2020.1771060

[ref61] Friedmann E. Son H. (2009). The human–companion animal bond: how humans benefit. Vet. Clin. N. Am. Small Anim. Pract. 39, 293–326. doi: 10.1016/j.cvsm.2008.10.015, PMID: 19185195

[ref62] Gergen P. J. Mitchell H. E. Calatroni A. Sever M. L. Cohn R. D. Salo P. M.. (2018). Sensitization and exposure to pets: the effect on asthma morbidity in the US population. The journal of allergy and clinical immunology. In Pract. 6, 101–107.e2. doi: 10.1016/j.jaip.2017.05.019, PMID: 28694047 PMC5756688

[ref63] Goldberg K. J. (2017). Exploring caregiver burden within a veterinary setting. Vet. Rec. 181, 318–319. doi: 10.1136/vr.j4156, PMID: 28923851

[ref64] Goleman M. Drozd L. Karpiński M. Czyżowski P. (2012). Cat therapy as an alternative form of animal-assisted therapy. Med. Weter. 26, 458–462. doi: 10.1111/j.1365-2915.2012.01017.x

[ref65] Gulick E. E. Krause-Parello C. A. (2012). Factors related to type of companion pet owned by older women. J. Psychosoc. Nurs. Ment. Health Serv. 50, 30–37. doi: 10.3928/02793695-20121003-01, PMID: 23066827

[ref66] Hajek A. König H. H. (2020). How do cat owners, dog owners and individuals without pets differ in terms of psychosocial outcomes among individuals in old age without a partner? Aging Ment. Health 24, 1613–1619. doi: 10.1080/13607863.2019.1647137 31364868

[ref67] Handlin L. Hydbring-Sandberg E. Nilsson A. Ejdebäck M. Jansson A. Uvnäs-Moberg K. (2011). Short-term interaction between dogs and their owners: effects on oxytocin, cortisol, insulin and heart rate—an exploratory study. Anthrozoös 24, 301–315. doi: 10.2752/175303711X13045914865385

[ref68] Harper C. M. Dong Y. Thornhill T. S. Wright J. Ready J. Brick G. W.. (2015). Can therapy dogs improve pain and satisfaction after total joint arthroplasty? A randomized controlled trial. Clin. Orthop. Relat. Res. 473, 372–379. doi: 10.1007/s11999-014-3931-0, PMID: 25201095 PMC4390934

[ref69] Hayama S. Chang L. Gumus K. King G. R. Ernst T. (2016). Neural correlates for perception of companion animal photographs. Neuropsychologia 85, 278–286. doi: 10.1016/j.neuropsychologia.2016.03.018, PMID: 27020140 PMC6502473

[ref70] Headey B. Na F. Zheng R. (2008). Pet dogs benefit owners’ health: a ‘natural experiment’in China. Soc. Indic. Res. 87, 481–493. doi: 10.1007/s11205-007-9142-2

[ref71] Heinrichs M. Baumgartner T. Kirschbaum C. Ehlert U. (2003). Social support and oxytocin interact to suppress cortisol and subjective responses to psychosocial stress. Biol. Psychiatry 54, 1389–1398. doi: 10.1016/s0006-3223(03)00465-7, PMID: 14675803

[ref72] Herrmann L. L. Ebmeier K. P. (2006). Factors modifying the efficacy of transcranial magnetic stimulation in the treatment of depression: a review. J. Clin. Psychiatry 67, 1870–1876. doi: 10.4088/jcp.v67n1206, PMID: 17194264

[ref73] Herrmann M. J. Ehlis A. C. Fallgatter A. J. (2004). Bilaterally reduced frontal activation during a verbal fluency task in depressed patients as measured by near-infrared spectroscopy. J. Neuropsychiatry Clin. Neurosci. 16, 170–175. doi: 10.1176/jnp.16.2.170, PMID: 15260368

[ref74] Himsworth C. G. Rock M. (2013). Pet ownership, other domestic relationships, and satisfaction with life among seniors: results from a Canadian national survey. Anthrozoös 26, 295–305. doi: 10.2752/175303713X13636846944448

[ref75] Hoagwood K. Vincent A. Acri M. Morrissey M. Seibel L. Guo F.. (2022). Reducing anxiety and stress among youth in a CBT-based equine-assisted adaptive riding program. Animals 12:2491. doi: 10.3390/ani12192491, PMID: 36230232 PMC9558534

[ref76] Ingram K. M. Cohen-Filipic J. (2019). Benefits, challenges, and needs of people living with cancer and their companion dogs: An exploratory study. J. Psychosoc. Oncol. 37, 110–126. doi: 10.1080/07347332.2018.1529010, PMID: 30592245

[ref77] Iwata M. Ota K. T. Duman R. S. (2013). The inflammasome: pathways linking psychological stress, depression, and systemic illnesses. Brain Behav. Immun. 31, 105–114. doi: 10.1016/j.bbi.2012.12.008, PMID: 23261775 PMC4426992

[ref78] Janak P. H. Tye K. M. (2015). From circuits to behaviour in the amygdala. Nature 517, 284–292. doi: 10.1038/nature14188, PMID: 25592533 PMC4565157

[ref79] Janssens M. Janssens E. Eshuis J. Lataster J. Simons M. Reijnders J.. (2021). Companion animals as buffer against the impact of stress on affect: an experience sampling study. Animals 11:2171. doi: 10.3390/ani11082171, PMID: 34438629 PMC8388427

[ref80] Julius H. Beetz A. Kotrschal K. Turner D. Uvnäs-Moberg K. (2012). Attachment to pets: An integrative view of human-animal relationships with implications for therapeutic practice. Hogrefe Publishing GmbH. Toronto

[ref81] Junça-Silva A. (2022). Friends with benefits: the positive consequences of pet-friendly practices for workers’ well-being. Int. J. Environ. Res. Public Health 19:1069. doi: 10.3390/ijerph19031069, PMID: 35162092 PMC8834589

[ref82] Junça-Silva A. Almeida M. Gomes C. (2022). The role of dogs in the relationship between telework and performance via affect: a moderated moderated mediation analysis. Animals 12:1727. doi: 10.3390/ani12131727, PMID: 35804626 PMC9264855

[ref83] Kidd A. H. Kidd R. M. (1998). Problems and benefits of bird ownership. Psychol. Rep. 83, 131–138. doi: 10.2466/pr0.1998.83.1.131

[ref84] Knight S. Edwards V. (2008). In the company of wolves: the physical, social, and psychological benefits of dog ownership. J. Aging Health 20, 437–455. doi: 10.1177/0898264308315875, PMID: 18448686

[ref85] Ko H. J. Youn C. H. Kim S. H. Kim S. Y. (2016). Effect of pet insects on the psychological health of community-dwelling elderly people: a single-blinded, randomized, controlled trial. Gerontology 62, 200–209. doi: 10.1159/000439129, PMID: 26383099

[ref86] Kobayashi A. Yamaguchi Y. Ohtani N. Ohta M. (2017). The effects of touching and stroking a cat on the inferior frontal gyrus in people. Anthrozoös 30, 473–486. doi: 10.1080/08927936.2017.1335115

[ref87] Kogan L. R. Currin-McCulloch J. Bussolari C. Packman W. Erdman P. (2021). The psychosocial influence of companion animals on positive and negative affect during the COVID-19 pandemic. Animals 11:2084. doi: 10.3390/ani11072084, PMID: 34359212 PMC8300185

[ref88] Kohn N. Eickhoff S. B. Scheller M. Laird A. R. Fox P. T. Habel U. (2014). Neural network of cognitive emotion regulation—an ALE meta-analysis and MACM analysis. NeuroImage 87, 345–355. doi: 10.1016/j.neuroimage.2013.11.001, PMID: 24220041 PMC4801480

[ref89] Kojima K. Brown E. C. Matsuzaki N. Asano E. (2013). Animal category-preferential gamma-band responses in the lower-and higher-order visual areas: intracranial recording in children. Clin. Neurophysiol. 124, 2368–2377. doi: 10.1016/j.clinph.2013.05.030, PMID: 23910987 PMC3834016

[ref90] Krause-Parello C. A. (2012). Pet ownership and older women: the relationships among loneliness, pet attachment support, human social support, and depressed mood. Geriatr. Nurs. 33, 194–203. doi: 10.1016/j.gerinurse.2011.12.005, PMID: 22321806

[ref91] Krause-Parello C. A. Gulick E. E. Basin B. (2019). Loneliness, depression, and physical activity in older adults: the therapeutic role of human–animal interactions. Anthrozoös 32, 239–254. doi: 10.1080/08927936.2019.1569906

[ref92] Lee J. H. Liu Q. Dadgar-Kiani E. (2022). Solving brain circuit function and dysfunction with computational modeling and optogenetic fMRI. Science 378, 493–499. doi: 10.1126/science.abq3868, PMID: 36327349 PMC10543742

[ref93] Lem M. Coe J. B. Haley D. B. Stone E. (2013). Effects of companion animal ownership among Canadian street-involved youth: a qualitative analysis. J. Soc. Soc. Welfare 40:285. doi: 10.15453/0191-5096.3771

[ref94] Leng M. Liu P. Zhang P. Hu M. Zhou H. Li G.. (2019). Pet robot intervention for people with dementia: a systematic review and meta-analysis of randomized controlled trials. Psychiatry Res. 271, 516–525. doi: 10.1016/j.psychres.2018.12.032, PMID: 30553098

[ref95] Liu X. Gao J. (2021). Application of animal-assisted intervention in the elderly population. Chin. J. Clin. Psych. 3, 656–660. doi: 10.16128/j.cnki.1005-3611.2021.03.043

[ref96] Liu W. Mao Y. Wei D. Yang J. Du X. Xie P.. (2016). Structural asymmetry of dorsolateral prefrontal cortex correlates with depressive symptoms: evidence from healthy individuals and patients with major depressive disorder. Neurosci. Bull. 32, 217–226. doi: 10.1007/s12264-016-0025-x, PMID: 27015663 PMC5563769

[ref97] Liu S. Powell L. Chia D. Russ T. C. McGreevy P. D. Bauman A. E.. (2019). Is dog ownership associated with mental health? A population study of 68, 362 adults living in England. Anthrozoös 32, 729–739. doi: 10.1080/08927936.2019.1673033

[ref98] Ma H. Jiang Y. Wang T. (2019). Research on equine-assisted intervention and autism Spectrum disorders, 2013-2018. Chin. J. Spec. Educ. 6, 37–46. doi: 10.3969/j.issn.1007-3728.2019.06.008

[ref99] MacDonald K. MacDonald T. M. (2010). The peptide that binds: a systematic review of oxytocin and its prosocial effects in humans. Harv. Rev. Psychiatry 18, 1–21. doi: 10.3109/10673220903523615 20047458

[ref9001] Mallon G. P. (1999). Animal-assisted therapy interventions with children. Innovative Psychotherapy Techniques in Child and Adolescent therapy, 2, 415–434.

[ref100] Marcus D. A. (2013). The science behind animal-assisted therapy. Curr. Pain Headache Rep. 17, 1–7. doi: 10.1007/s11916-013-0322-2, PMID: 23430707

[ref101] Martens P. Hansart C. Su B. (2019). Attitudes of young adults toward animals—the case of high school students in Belgium and the Netherlands. Animals 9:88. doi: 10.3390/ani9030088, PMID: 30862099 PMC6466541

[ref103] Marusak H. A. Martin K. R. Etkin A. Thomason M. E. (2015). Childhood trauma exposure disrupts the automatic regulation of emotional processing. Neuropsychopharmacology 40, 1250–1258. doi: 10.1038/npp.2014.311, PMID: 25413183 PMC4367470

[ref104] Matamoros O. M. del Villar E. Y. A. Padilla R. T. (2022). Analysis of EEG signals in a patient with spastic cerebral palsy undergone dolphin-assisted therapies. Int. J. Adv. Comput. Sci. Appl. 13, 918–929. doi: 10.14569/IJACSA.2022.01312106

[ref105] Matsumura K. Hamazaki K. Tsuchida A. Inadera H. (2022). Pet ownership during pregnancy and mothers’ mental health conditions up to 1 year postpartum: a nationwide birth cohort—the Japan environment and Children’s study. Soc. Sci. Med. 309:115216. doi: 10.1016/j.socscimed.2022.115216, PMID: 36029711

[ref106] Matsuura A. Aiba N. Yamamoto H. Takahashi M. Kido H. Suzuki T.. (2020). Stroking a real horse versus stroking a toy horse: effects on the frontopolar area of the human brain. Anthrozoös 33, 673–683. doi: 10.1080/08927936.2020.1799564

[ref107] McConnell A. R. Brown C. M. Shoda T. M. Stayton L. E. Martin C. E. (2011). Friends with benefits: on the positive consequences of pet ownership. J. Pers. Soc. Psychol. 101, 1239–1252. doi: 10.1037/a0024506, PMID: 21728449

[ref108] Meixner J. Kotrschal K. (2022). Animal-assisted interventions with dogs in special education—a systematic review. Front. Psychol. 13:876290. doi: 10.3389/fpsyg.2022.876290, PMID: 35712211 PMC9197485

[ref109] Melson G. F. (2003). Child development and the human-companion animal bond. Am. Behav. Sci. 47, 31–39. doi: 10.1177/0002764203255210

[ref110] Menna L. F. Santaniello A. Gerardi F. Di Maggio A. Milan G. (2016). Evaluation of the efficacy of animal-assisted therapy based on the reality orientation therapy protocol in Alzheimer’s disease patients: a pilot study. Psychogeriatrics 16, 240–246. doi: 10.1111/psyg.12145, PMID: 26370064

[ref111] Messam L. L. M. Hart L. A. (2019). “Persons experiencing prolonged grief after the loss of a pet” in Clinician’s guide to treating companion animal issues. eds. Kogan L. R. Blazina C. (Cambridge, MA: Academic Press), 267–280.

[ref112] Miles J. N. Parast L. Babey S. H. Griffin B. A. Saunders J. M. (2017). A propensity-score-weighted population-based study of the health benefits of dogs and cats for children. Anthrozoös 30, 429–440. doi: 10.1080/08927936.2017.1335103

[ref113] Miller M. Lago D. (1990). The well-being of older women: the importance of pet and human relations. Anthrozoös 3, 245–252. doi: 10.2752/089279390787057504

[ref114] Miltiades H. Shearer J. (2011). Attachment to pet dogs and depression in rural older adults. Anthrozoös 24, 147–154. doi: 10.2752/175303711X12998632257585

[ref115] Moretti F. De Ronchi D. Bernabei V. Marchetti L. Ferrari B. Forlani C.. (2011). Pet therapy in elderly patients with mental illness. Psychogeriatrics 11, 125–129. doi: 10.1111/j.1479-8301.2010.00329.x 21707862

[ref116] Mormann F. Dubois J. Kornblith S. Milosavljevic M. Cerf M. Ison M.. (2011). A category-specific response to animals in the right human amygdala. Nat. Neurosci. 14, 1247–1249. doi: 10.1038/nn.2899, PMID: 21874014 PMC3505687

[ref117] Mossello E. Ridolfi A. Mello A. M. Lorenzini G. Mugnai F. Piccini C.. (2011). Animal-assisted activity and emotional status of patients with Alzheimer’s disease in day care. Int. Psychogeriatr. 23, 899–905. doi: 10.1017/S1041610211000226, PMID: 21356158

[ref118] Myrick J. G. (2015). Emotion regulation, procrastination, and watching cat videos online: who watches internet cats, why, and to what effect? Comput. Hum. Behav. 52, 168–176. doi: 10.1016/j.chb.2015.06.001

[ref119] Na H. Dong S. Y. (2023). Mixed-reality-based human-animal interaction can relieve mental stress. Front. Vet. Sci. 10:1102937. doi: 10.3389/fvets.2023.1102937, PMID: 37008360 PMC10060814

[ref120] Nagasawa M. Kikusui T. Onaka T. Ohta M. (2009). Dog’s gaze at its owner increases owner’s urinary oxytocin during social interaction. Horm. Behav. 55, 434–441. doi: 10.1016/j.yhbeh.2008.12.002, PMID: 19124024

[ref121] Nagasawa T. Kimura Y. Masuda K. Uchiyama H. (2023). Effects of interactions with cats in domestic environment on the psychological and physiological state of their owners: associations among cortisol, oxytocin, heart rate variability, and emotions. Animals 13:2116. doi: 10.3390/ani13132116, PMID: 37443915 PMC10340037

[ref122] Nater U. M. Skoluda N. Strahler J. (2013). Biomarkers of stress in behavioural medicine. Curr. Opin. Psychiatry 26, 440–445. doi: 10.1097/YCO.0b013e328363b4ed 23867656

[ref124] Nie J. (2020). Research on “raising pets by clouds” from the perspective of youth subculture (Master’s thesis). Hunan Normal University. Changsha

[ref125] Nimer J. Lundahl B. (2007). Animal-assisted therapy: a meta-analysis. Anthrozoös 20, 225–238. doi: 10.2752/089279307X224773

[ref126] O’Callaghan D. M. (2008). Exploratory study of animal assisted therapy interventions used by mental health professionals University of North Texas. Denton, TX

[ref127] Odendaal J. S. Lehmann S. M. C. (2000). The role of phenylethylamine during positive human-dog interaction. Acta Vet. Brno 69, 183–188. doi: 10.2754/avb200069030183

[ref128] Odendaal J. S. Meintjes R. A. (2003). Neurophysiological correlates of affiliative behaviour between humans and dogs. Vet. J. 165, 296–301. doi: 10.1016/S1090-0233(02)00237-X, PMID: 12672376

[ref129] O’Haire M. E. (2013). Animal-assisted intervention for autism spectrum disorder: a systematic literature review. J. Autism Dev. Disord. 43, 1606–1622. doi: 10.1007/s10803-012-1707-5, PMID: 23124442

[ref130] O’Haire M. E. McKenzie S. J. Beck A. M. Slaughter V. (2013). Social behaviors increase in children with autism in the presence of animals compared to toys. PLoS One 8:e57010. doi: 10.1371/journal.pone.0057010, PMID: 23468902 PMC3584132

[ref131] Oliva J. L. Johnston K. L. (2021). Puppy love in the time of Corona: dog ownership protects against loneliness for those living alone during the COVID-19 lockdown. Int. J. Soc. Psychiatry 67, 232–242. doi: 10.1177/0020764020944195 32701015 PMC7383093

[ref132] Ortmeyer H. K. Giffuni J. Etchberger D. Katzel L. (2023). The role of companion dogs in the VA Maryland health care system whole health (y) Gero fit program. Animals 13:3047. doi: 10.3390/ani13193047, PMID: 37835653 PMC10571922

[ref133] Ouanes S. Popp J. (2019). High cortisol and the risk of dementia and Alzheimer’s disease: a review of the literature. Front. Aging Neurosci. 11:43. doi: 10.3389/fnagi.2019.00043, PMID: 30881301 PMC6405479

[ref134] Pagani C. Robustelli F. Ascione F. R. (2007). Italian youths’ attitudes toward, and concern for, animals. Anthrozoös 20, 275–293. doi: 10.2752/089279307X224818

[ref135] Peacock J. Chur-Hansen A. Winefield H. (2012). Mental health implications of human attachment to companion animals. J. Clin. Psychol. 68, 292–303. doi: 10.1002/jclp.20866, PMID: 22307948

[ref136] Pennacchio S. Tripi G. Salerno M. Russo D. Lavano S. Cerroni F.. (2018). Animals-assisted therapy: a brief review. Acta Medica Mediterranea 34, 2089–2095. doi: 10.19193/0393-6384-2018-4s-323

[ref137] Peretti P. O. (1990). Elderly-animal friendship bonds. Soc. Behav. Personal. Int. J. 18, 151–156. doi: 10.2224/sbp.1990.18.1.151

[ref138] Pikhartova J. Bowling A. Victor C. (2014). Does owning a pet protect older people against loneliness? BMC Geriatr. 14:106. doi: 10.10.1186/1471-2318-14-106, PMID: 25240250 PMC4182770

[ref139] Pivonello R. De Martino M. C. Iacuaniello D. Simeoli C. Muscogiuri G. Carlomagno F.. (2016). Metabolic alterations and cardiovascular outcomes of cortisol excess. Front. Horm. Res. 46, 54–65. doi: 10.1159/000443864, PMID: 27212264

[ref140] Polheber J. P. Matchock R. L. (2014). The presence of a dog attenuates cortisol and heart rate in the trier social stress test compared to human friends. J. Behav. Med. 37, 860–867. doi: 10.1007/s10865-013-9546-1, PMID: 24170391

[ref141] Pollak C. Wexler S. S. Drury L. (2022). Effect of a robotic pet on social and physical frailty in community-dwelling older adults: a randomized controlled trial. Res. Gerontol. Nurs. 15, 229–237. doi: 10.3928/19404921-20220830-01, PMID: 36113009

[ref142] Pressman P. Rosen H. J. (2015). “Disorders of frontal lobe function” in Neurobiology of Brain Disorders. eds. Zigmond M. J. Rowland L. P. Coyle J. T. (Amsterdam: Elsevier), 542–557.

[ref143] Prieto A. Martins Almeida Ayupe K. Nemetala Gomes L. Saúde A. C. Gutierres Filho P. (2022). Effects of equine-assisted therapy on the functionality of individuals with disabilities: systematic review and meta-analysis. Physiother. Theory Pract. 38, 1091–1106. doi: 10.1080/09593985.2020.1836694, PMID: 33084452

[ref144] Purewal R. Christley R. Kordas K. Joinson C. Meints K. Gee N.. (2017). Companion animals and child/adolescent development: a systematic review of the evidence. Int. J. Environ. Res. Public Health 14:234. doi: 10.3390/ijerph14030234, PMID: 28264460 PMC5369070

[ref145] Randler C. Ballouard J. M. Bonnet X. Chandrakar P. Pati A. K. Medina-Jerez W.. (2021). Attitudes toward animal welfare among adolescents from Colombia, France, Germany, and India. Anthrozoös 34, 359–374. doi: 10.1080/08927936.2021.1898212

[ref146] Rathish D. Rajapakse J. Weerakoon K. (2022). “In times of stress, it is good to be with them”: experience of dog owners from a rural district of Sri Lanka. BMC Public Health 22, 2380–2312. doi: 10.1186/s12889-022-14863-6, PMID: 36536373 PMC9761628

[ref147] Rémillard L. W. Meehan M. P. Kelton D. F. Coe J. B. (2017). Exploring the grief experience among callers to a pet loss support hotline. Anthrozoös 30, 149–161. doi: 10.1080/08927936.2017.1270600

[ref148] Riddick C. C. (1985). Health, aquariums, and the non-institutionalized elderly. Marriage Fam. Rev. 8, 163–173. doi: 10.1300/J002v08n03_12

[ref149] Russell E. Koren G. Rieder M. Van Uum S. (2012). Hair cortisol as a biological marker of chronic stress: current status, future directions and unanswered questions. Psychoneuroendocrinology 37, 589–601. doi: 10.1016/j.psyneuen.2011.09.009 21974976

[ref150] Sable P. (2013). The pet connection: an attachment perspective. Clin. Soc. Work. J. 41, 93–99. doi: 10.1007/s10615-012-0405-2

[ref151] Sander D. Grafman J. Zalla T. (2003). The human amygdala: an evolved system for relevance detection. Rev. Neurosci. 14, 303–316. doi: 10.1515/REVNEURO.2003.14.4.303, PMID: 14640318

[ref152] Sanders S. (2021). 125 questions: exploration and discovery. Science/AAAS Custom Publishing Office: Washington, DC, USA.

[ref153] Schoorlemmer R. M. M. Peeters G. M. E. E. Van Schoor N. M. Lips P. T. A. M. (2009). Relationships between cortisol level, mortality and chronic diseases in older persons. Clin. Endocrinol. 71, 779–786. doi: 10.1111/j.1365-2265.2009.03552.x, PMID: 19226268

[ref154] Schwarzmüller-Erber G. Maier M. Stummer H. Kundi M. (2021). Recreational horseback riding and its association with physical, mental, and social wellbeing and perceived health. Anthrozoös 34, 685–706. doi: 10.1080/08927936.2021.1926709

[ref155] Shearer A. Hunt M. Chowdhury M. Nicol L. (2016). Effects of a brief mindfulness meditation intervention on student stress and heart rate variability. Int. J. Stress. Manag. 23, 232–254. doi: 10.1037/a0039814

[ref156] Shiloh S. Sorek G. Terkel J. (2003). Reduction of state-anxiety by petting animals in a controlled laboratory experiment. Anxiety Stress Coping 16, 387–395. doi: 10.1080/1061580031000091582

[ref157] Shin L. M. Liberzon I. (2010). The neurocircuitry of fear, stress, and anxiety disorders. Neuropsychopharmacology 35, 169–191. doi: 10.1038/npp.2009.83, PMID: 19625997 PMC3055419

[ref159] Solomon J. Beetz A. Schöberl I. Gee N. Kotrschal K. (2019). Attachment security in companion dogs: adaptation of Ainsworth’s strange situation and classification procedures to dogs and their human caregivers. Attach Hum. Dev. 21, 389–417. doi: 10.1080/14616734.2018.1517812, PMID: 30246604 PMC6532729

[ref160] Souter M. A. Miller M. D. (2007). Do animal-assisted activities effectively treat depression? A meta-analysis. Anthrozoös 20, 167–180. doi: 10.2752/175303707X207954

[ref161] Spitznagel M. B. Jacobson D. M. Cox M. D. Carlson M. D. (2017). Caregiver burden in owners of a sick companion animal: a cross-sectional observational study. Vet. Rec. 181:321. doi: 10.1136/vr.104295, PMID: 28870976

[ref162] Staufenbiel S. M. Penninx B. W. Spijker A. T. Elzinga B. M. van Rossum E. F. (2013). Hair cortisol, stress exposure, and mental health in humans: a systematic review. Psychoneuroendocrinology 38, 1220–1235. doi: 10.1016/j.psyneuen.2012.11.015, PMID: 23253896

[ref163] Stoeckel L. E. Palley L. S. Gollub R. L. Niemi S. M. Evins A. E. (2014). Patterns of brain activation when mothers view their own child and dog: an fMRI study. PLoS One 9:e107205. doi: 10.1371/journal.pone.0107205, PMID: 25279788 PMC4184794

[ref164] Sugawara A. Masud M. M. Yokoyama A. Mizutani W. Watanuki S. Yanai K.. (2012). Effects of presence of a familiar pet dog on regional cerebral activity in healthy volunteers: a positron emission tomography study. Anthrozoös 25, 25–34. doi: 10.2752/175303712X13240472427311

[ref165] Teo J. T. Thomas S. J. (2019). Psychological mechanisms predicting wellbeing in pet owners: Rogers’ core conditions versus Bowlby’s attachment. Anthrozoös 32, 399–417. doi: 10.1080/08927936.2019.1598660

[ref166] Thiebaut de Schotten M. Forkel S. J. (2022). The emergent properties of the connected brain. Science 378, 505–510. doi: 10.1126/science.abq2591 36378968

[ref167] Tower R. B. Nokota M. (2006). Pet companionship and depression: results from a United States internet sample. Anthrozoös 19, 50–64. doi: 10.2752/089279306785593874

[ref168] Trammell J. P. (2017). The effect of therapy dogs on exam stress and memory. Anthrozoös 30, 607–621. doi: 10.1080/08927936.2017.1370244

[ref169] Tseng S. H. Chen H. C. Tam K. W. (2013). Systematic review and meta-analysis of the effect of equine assisted activities and therapies on gross motor outcome in children with cerebral palsy. Disabil. Rehabil. 35, 89–99. doi: 10.3109/09638288.2012.687033, PMID: 22630812

[ref170] Tsigos C. Chrousos G. P. (2002). Hypothalamic–pituitary–adrenal axis, neuroendocrine factors and stress. J. Psychosom. Res. 53, 865–871. doi: 10.1016/s0022-3999(02)00429-4 12377295

[ref172] Virues-Ortega J. Pastor-Barriuso R. Castellote J. M. Poblacion A. de Pedro-Cuesta J. (2012). Effect of animal-assisted therapy on the psychological and functional status of elderly populations and patients with psychiatric disorders: a meta-analysis. Health Psychol. Rev. 6, 197–221. doi: 10.1080/17437199.2010.534965

[ref173] Vives A. H. De Angel V. Papadopoulos A. Strawbridge R. Wise T. Young A. H.. (2015). The relationship between cortisol, stress and psychiatric illness: new insights using hair analysis. J. Psychiatr. Res. 70, 38–49. doi: 10.1016/j.jpsychires.2015.08.007, PMID: 26424422

[ref174] Wang Y. Liu C. (2021). Research Progress of canine-assisted intervention in individuals with autism Spectrum disorders. Chin. J. Spec. Educ. 5, 37–43. doi: 10.3969/j.issn.1007-3728.2021.05.005

[ref175] Wells D. L. (2005). The effect of videotapes of animals on cardiovascular responses to stress. Stress. Health 21, 209–213. doi: 10.1002/smi.1057

[ref176] Wells D. L. (2009). The effects of animals on human health and well-being. J. Soc. Issues 65, 523–543. doi: 10.1111/j.1540-4560.2009.01612.x

[ref177] Wells D. L. (2019). The state of research on human–animal relations: implications for human health. Anthrozoös 32, 169–181. doi: 10.1080/08927936.2019.1569902

[ref178] Wilks K. (1999). When dogs are man’s best friend—the health benefits of companion animals in the modern society. Urban animal management (UAM) conference proceedings Gold Coast. Farnham: AVA Ltd.

[ref9002] Williams J. Lawrence A. Muldoon J. (2010). Children and their pets: exploring the relationships between ownership attitudes attachment empathy. Education and Health 28, 12–15.

[ref179] Wong P. W. C. Lau K. C. T. Liu L. L. Yuen G. S. N. Wing-Lok P. (2017). Beyond recovery: understanding the postbereavement growth from companion animal loss. Omega 75, 103–123. doi: 10.1177/0030222815612603 28490281

[ref180] Yaribeygi H. Panahi Y. Sahraei H. Johnston T. P. Sahebkar A. (2017). The impact of stress on body function: a review. EXCLI J. 16, 1057–1072. doi: 10.17179/excli2017-480 28900385 PMC5579396

[ref181] Yatcilla J. K. (2021). A panorama of human–animal interactions research: bibliometric analysis of HAI articles 1982–2018. Anthrozoös 34, 161–173. doi: 10.1080/08927936.2021.1885139

[ref182] Zalaf A. Egan V. (2015). A new questionnaire examining general attitudes toward animals in Cyprus and the United Kingdom. J. Vet. Behav. 10, 111–117. doi: 10.1016/j.jveb.2014.09.003

[ref183] Zalaf A. Egan V. (2017). Cyprus versus UK: cultural differences of attitudes toward animals based on personality and sensational interests. Anthrozoös 30, 47–60. doi: 10.1080/08927936.2017.1270592

[ref184] Zhou Z. Yin D. Gao Q. (2020). Sense of presence and subjective well-being in online pet watching: the moderation role of loneliness and perceived stress. Int. J. Environ. Res. Public Health 17:9093. doi: 10.3390/ijerph17239093, PMID: 33291458 PMC7730746

[ref185] Zhou Q. Zhu Y. Hong Q. (2019). Research progress on equine-assisted interventions in autism spectrum disorder children. Chinese. J. Child Health Care 8, 861–863+872. doi: 10.11852/zgetbjzz2019-0167

[ref187] Zilcha-Mano S. Mikulincer M. Shaver P. R. (2011). An attachment perspective on human–pet relationships: conceptualization and assessment of pet attachment orientations. J. Res. Pers. 45, 345–357. doi: 10.1016/j.jrp.2011.04.001

